# Multifaceted activity of millipede secretions: Antioxidant, antineurodegenerative, and anti-*Fusarium* effects of the defensive secretions of *Pachyiulus hungaricus* (Karsch, 1881) and *Megaphyllum unilineatum* (C. L. Koch, 1838) (Diplopoda: Julida)

**DOI:** 10.1371/journal.pone.0209999

**Published:** 2019-01-03

**Authors:** Bojan Ilić, Nikola Unković, Aleksandar Knežević, Željko Savković, Milica Ljaljević Grbić, Jelena Vukojević, Zvezdana Jovanović, Slobodan Makarov, Luka Lučić

**Affiliations:** 1 Department of Animal Development, University of Belgrade—Faculty of Biology, Belgrade, Serbia; 2 Department of Algology, Mycology, and Lichenology, University of Belgrade—Faculty of Biology, Belgrade, Serbia; College of Agricultural Sciences, UNITED STATES

## Abstract

Members of the millipede order Julida rely on dominantly quinonic defensive secretions with several minor, non-quinonic components. The free radical-scavenging activities of ethanol, methanol, hexane, and dichloromethane extracts of defensive secretions emitted by *Pachyiulus hungaricus* (Karsch, 1881) and *Megaphyllum unilineatum* (C. L. Koch, 1838) were investigated using the ABTS, DPPH, and total reducing power (TRP) tests. The obtained extracts were also tested for inhibition of acetylcholinesterase and tyrosinase activity. Finally, the antifungal potential of both julid extracts was evaluated against seven *Fusarium* species. Secretions of both species showed activity against free radicals, acetylcholinesterase, tyrosinase, and all of the selected fungal species. The secretions of *P*. *hungaricus* exhibited a more potent antioxidative effect than did those of *M*. *unilineatum*, while there were no significant differences of antiacetylcholinesterase activity between the tested extracts. Only the hexane extract of *M*. *unilineatum* showed an effect on tyrosinase activity stronger than that of *P*. *hungaricus*. *Fusarium sporotrichioides*, *F*. *graminearum*, and *F*. *verticillioides* were the fungi most resistant to secretions of both julids. The *Fusarium* species most susceptible to the secretion of *P*. *hungaricus* was *F*. *avenaceum*, while the concentrations of *M*. *unilienatum* extracts needed to inhibit and completely suppress fungal growth were lowest in the case of their action on *F*. *lateritium*. Our data support previous findings that julid defensive secretions possess an antimicrobial potential and reveal their antioxidative and antineurodegenrative properties. Bearing in mind the chemical complexity of the tested defensive secretions, we presume that they can also exhibit other biological activities.

## Introduction

With more than 1.2 million species [1], arthropods are arguably the most speciose and abundant group in the animal kingdom. Their impressive and unique evolutionary success is reflected in the remarkably great morphological, physiological, developmental and ecological diversity of arthropods [2]. However, there is at least one more cause of their evolutionary success. The highly variable biology of arthropods is matched by diverse and complex antipredator defences present in these invertebrates [3]. It is presumed that the evolution of chemical defences played an important role in the early diversification of arthropods [4].

**Fig 1:**
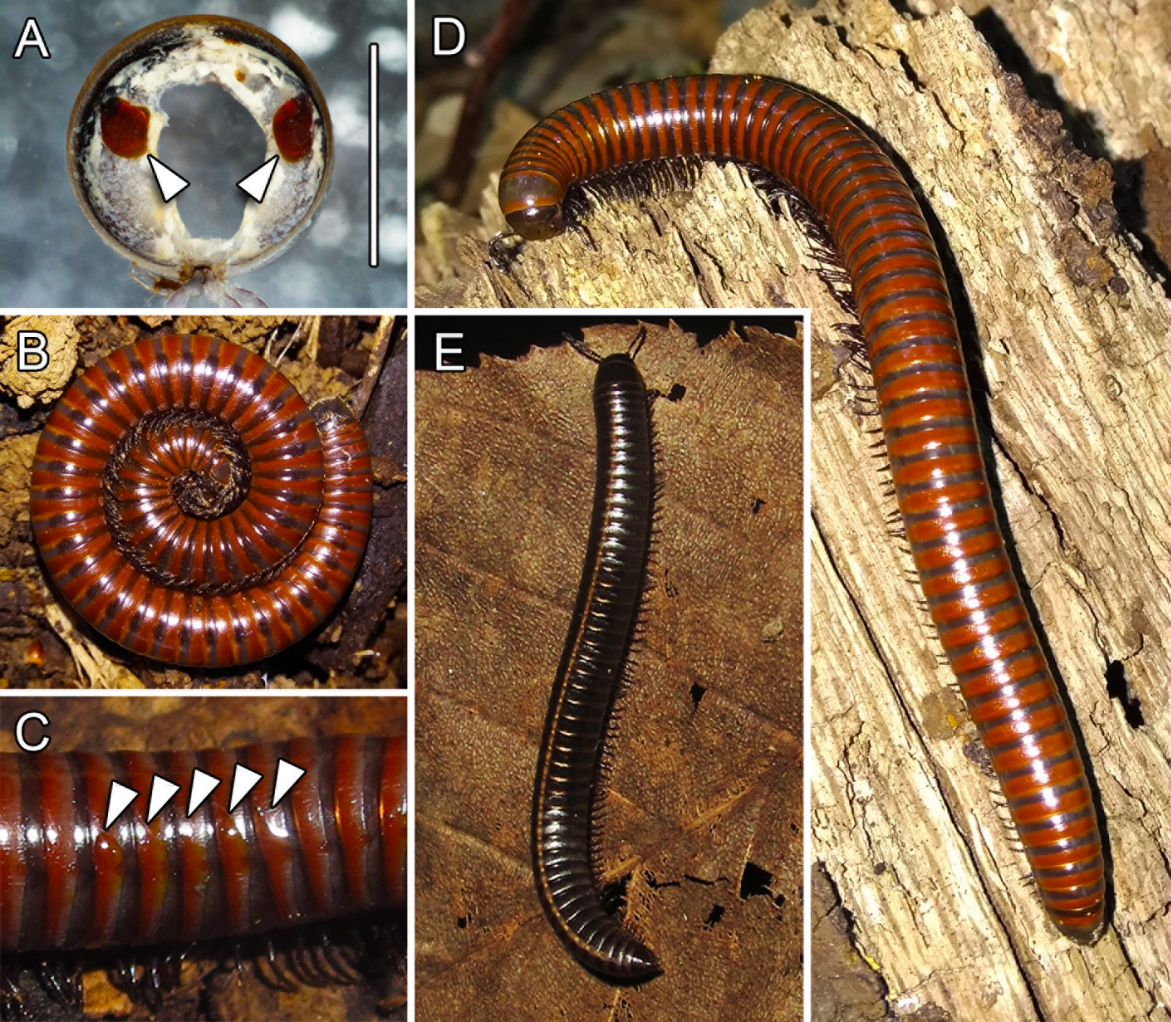
Segment of *Megaphyllum unilineatum* with pair of defensive glands (arrowheads) (A); spiral coiling of *Pachyiulus hungaricus* (B); close-up of *P*. *hungaricus* trunk with droplets of defensive secretions (arrowheads) (C); *P*. *hungaricus* (D); *M*. *unilineatum* (E). Scale bar = 2 mm.

Although the first body fossils of millipedes (Diplopoda) can be traced to the Silurian, molecular data indicate that their diversification dates from the Middle Ordovician or earliest Silurian [5–7]. Hence, this group of animals is among the oldest of terrestrial arthropods. Diplopods constitute the largest class within the subphylum Myriapoda, and they are distributed on all continents (except Antarctica) [8,9]. Millipedes are soil and litter dwellers with an important ecological role in the decomposition of rotting plant material, thereby contributing to the cycling of several chemical elements [10]. One of the most conspicuous peculiarities of millipedes is the presence of defensive glands (ozadenes) along the trunk (Fig 1A). These exocrine organs are segmentally arranged and variable, both in morpho-anatomic features and in the nature of secreted chemicals. Based on their architecture, there are four types of millipede defensive glands–glomerid, julid, polydesmid, and colobognathan [11–13]. In the event of attack by a predator, most millipedes coil into a spiral with the head at the centre of the spiral (Fig 1B), exposing laterally located defensive gland openings (ozopores) and releasing secretions toward the exterior (Fig 1C). The major compounds secreted by millipedes can be roughly listed as quinones, phenolics, cyanogenetic compounds, and alkaloids [12,14].

One of the hallmarks of the millipede order Julida (as well as their closest relatives Spirobolida and Spirostreptida) is the synthesis of quinone-based defensive secretions [14–17]. Exudates of julidan defensive glands are cocktails made of different compounds–benzoquinones, phenolics, alcohols, aldehydes, esters, anthranilate derivatives, and ketones ([16] and references therein). The number of compounds in these secretions can vary from two to ca. 60 [4,18]. These facts mean that julidan defensive chemistry is the most complex within the Diplopoda [4].

Due to the presence of diverse chemical compounds in julid defensive secretions, it seems reasonable to assume that these exudates can show different biological activities. So far, defensive secretions of four julid species (*Cylindroiulus boleti* (C. L. Koch, 1847), *Megaphyllum bosniense* (Verhoeff, 1897), *M*. *unilineatum* (C. L. Koch, 1838), and *Pachyiulus hungaricus* (Karsch, 1881)) have been tested against different bacterial and fungal pathogens [18,19]. Both studies revealed that the tested julid defensive secretions are highly potent antifungals and moderately strong antibacterials. In order to explore and expand our knowledge about the antifungal potential of these exudates, we evaluated properties of defensive secretions of the latter two mentioned species against seven *Fusarium* species. *Fusarium* spp. are important pathogens of plants and mammals (including humans) which exhibit moderate to high resistance to many commercial used antifungal agents [20,21]. Based on the known chemical nature of selected defensive secretions [18,19], we also designed this study to gain insight into the antioxidative and antineurodegenrative potential of these two julid secretions.

## Materials and methods

### Collecting and handling of millipedes

Adult individuals of *Pachyiulus hungaricus* (Fig 1D) were collected during October of 2017 on Mt. Avala (N 44°41′32″; E 20°31′06″) near Belgrade, Serbia, while adults of *Megaphyllum unilineatum* (Fig 1E) were collected during the same period in the Krnjača suburb of Belgrade (N 44°50′29″; E 20°29′05″). After capture, the millipedes were kept in plastic boxes containing ground cover from the collecting site. The boxes were regularly sprayed with water to maintain high humidity. Due to the fact that the sample was female-biased, defensive secretions of female specimens were used for further analyses. Animals used in our study are not endangered and/or protected species and specific permissions were not required for their collecting on above mentioned localities. All individuals used in this study are deposited in the collection of the Institute of Zoology, University of Belgrade–Faculty of Biology, Belgrade, Serbia.

### Preparation of defensive secretions of *P*. *hungaricus* and *M*. *unilineatum*

Excretion of defensive secretions was elicited from glands of five *P*. *hungaricus* and 100 *M*. *unilineatum* females via mechanical stress in closed glass vials. Secretions collected from both species were dissolved in 5 mL and 10 mL of 96% ethanol (EtOH), methanol (MetOH), hexane (Hex), and dichloromethane (DCM), respectively, concentrated under reduced pressure in a rotary evaporator (Rotavapor R-210, Buchi) at 40°C to a dry residues, and redissolved in a given solvent (due to high volatility, dimethyl sulphoxide (DMSO) was used in lieu of Hex and DCM). The final concentration of secretions used to test antifungal and antioxidative activities was 5 mg/mL.

### Determination of antioxidative activity

Antioxidative activities of extracts of the defensive secretions of *P*. *hungaricus* and *M*. *unilineatum* were determined by three methods, viz., the ABTS, DPPH, and total reducing power (TRP) tests.

### ABTS assay

The ABTS method involves direct generation of the ABTS^+**●**^ chromophore through the reaction between ABTS and potassium persulphate (K_2_S_2_O_8_). The method is based on measuring the change in colour of an ABTS^+**●**^ stock solution in the presence of antioxidants according to the procedure of Miller et al. [22]. The initial solution of ABTS cation radicals was prepared by dissolving 9.0 μg of ABTS in 2.5 mL of dH_2_O and adding 44.0 μL of a 140 mM K_2_S_2_O_8_ solution 12 to 16 hours before the start of the experiment, while the stock solution was prepared immediately prior to measurement by diluting the initial solution with dH_2_O and adjusting the solution’s absorbance by 0.700 ± 0.020 at 734 nm. The reaction mixture (1500.0 μL of the ABTS stock solution and 15.0 μL of extract with a concentration of 1.0 mg/mL) was incubated at room temperature for 4 min and the absorbance change was measured spectrophotometrically at 734 nm. Distilled water was used as a blank. The extract concentration required for ABTS^+●^ reduction, equivalent to reduction of 1.0 mg/mL ascorbic acid (AAEC), was determined using the equation of the calibration curve for ascorbic acid. The EC_50_ value (mg extract/mL) represents the effective extract concentration that scavenges 50% of ABTS cation radicals and is obtained by linear regression analysis. Each sample was measured in triplicate and averaged.

### DPPH assay

Determination of free radical-scavenging activity was based on bleaching of the purple-coloured methanol solution of the stable 1,1-diphenyl-2-picryl-hydrazil (DPPH**^●^**) radical [23]. Reaction mixtures [1800 μL of a 4% (w/v) methanol solution of DPPH**^●^** and 200 μL of the defined concentration of extract] were incubated in the dark on a rotary shaker (100 rpm) for 30 min. Scavenging effects were measured spectrophotometrically (CECIL CE 2501) at 517 nm against methanol as the blank and calculated using the equation:

$${\mathrm{DPPH}}^{●}‑\mathrm{scavenging}\;\mathrm{effect}\;(\%)=[(A_{0}‑A_{\mathrm{sample}})\;A_{0}^{‑1}]\times 100,$$

where A_0_ –is absorbance of the negative control (reaction mixture without extract); and A_sample_−is absorbance of the reaction mixture.

The extract concentration (mg extract/mL) providing 50% of DPPH**^●^** reduction (EC_50_) was obtained by interpolation from linear regression analysis. All measurements were carried out in triplicate for statistical analysis. A commercial antioxidant, butylated hydroxyanisole (BHA), was used as a positive control.

### Reducing power assay

The reducing power assay was based on the ability of antioxidative compounds to reduce potassium ferricyanide (K_3_[Fe(CN)_6_]) to potassium ferrocyanide (K_4_[Fe(CN)_6_]), a blue-coloured complex [24]. The reaction mixture containing 1.0 mL of extract (at a defined concentration ranging from 0.1 mg/mL to 2.5 mg/mL), 2.5 mL of 0.2 M sodium phosphate buffer (pH 6.6), and 2.5 mL of 1% (w/v) K_3_[Fe(CN)_6_] was incubated in a water bath (Memmert WB7) at 5°C for 20 min. After cooling on ice, 2.5 mL of a 10% (w/v) CCl_3_COOH solution was added and the mixture was centrifuged at 3000 rpm for 10 min. A 2.5-mL aliquot of the upper layer was mixed with 2.5 mL of dH_2_O and 1.0 mL of a 0.1% (w/v) FeCl_3_ solution, and the absorbance was recorded spectrophotometrically (CECIL CE2501 BioQuest) at 700 nm. Higher absorbance indicated higher reducing power. A mixture containing K_3_[Fe(CN)_6_], sodium phosphate buffer, and a reference solvent was used as a blank. A standard curve of ascorbic acid (y = 1.737x+0.034, R^2^ = 0.999) was prepared and results were expressed as milligrams of ascorbic acid equivalents (AAE) per milligram of dried extract. The assays were carried out in triplicate and the results are expressed as mean values ± standard errors.

### Antineurodegenerative activity assays

#### Determination of acetylcholinesterase inhibitory activity

The rate of inhibition of acetylcholinesterase (AChE) activity was determined spectrophotometrically using 96-well microtitre plates employing the method of Ellman et al. [25]. Acetylcholine iodide (AChI) was used as substrate for the enzyme AChE, which degrades this compound to acetate and thiocholine. In the next step, 5,5′-dithiobis(2-nitrobenzoic acid) (DTNB) was transformed with thiocholine to the yellow-coloured 5-thio-2-nitrobenzoate anion (TNB^2-^) and the change in absorbance was recorded at a wavelength of 412 nm and temperature of 25°C. The reaction mixture contained 140.0 μL of 0.1 mM sodium phosphate buffer (pH 8.0), 20.0 μL of DTNB, 20.0 μL of extract (100 μg/mL), 20.0 μL of AChE, and 10.0 μL of AChI. A mixture of 5% (v/v) DMSO and sodium phosphate buffer (pH 8.0) was used as a blank. The rate of inhibition of AChE activity was determined according to the formula:

$$\mathrm{Rate}\;\mathrm{of}\;\mathrm{inhibition}\;\mathrm{of}\;\mathrm{AChE}\;\mathrm{activity}\;(\%)=\left[\frac{E‑S}{E}\right]\times 100$$

where E is enzyme activity without the extract and S is enzyme activity with the extract.

The obtained values were compared with a commercial inhibitor of AChE, viz., galantamine.

#### Determination of tyrosinase inhibitory activity

The rate of inhibition of tyrosinase activity was determined spectrophotometrically using 96-well microtitre plates by the method of Likhitwitayawuid and Sritularak [26]. The reaction mixture (80.0 μL of 66.7 mM phosphate buffer (pH 6.8), 40.0 μL of extract (100 μg/mL), and 40.0 μL of tyrosinase (46 U/L) dissolved in phosphate buffer) was incubated at 23°C for 10 min, after which 40.0 μL of 2.5 mM L-DOPA was added. Absorbance was measured after 30 min of incubation at 475 nm for dopachrome formation in the reaction mixture. The rate of inhibition of tyrosinase activity was determined according to the formula:

$$\mathrm{Rate}\;\mathrm{of}\;\mathrm{inhibition}\;\mathrm{of}\;\mathrm{tyrosinase}\;\mathrm{activity}\;(\%)=\left[\frac{\left(A‑B)‑(C‑D\right)}{A‑B}\right]\times 100$$

where A is absorbance of tyrosinase in phosphate buffer, B is absorbance of phosphate buffer, C is absorbance of the reaction mixture, and D is absorbance of the extract in phosphate buffer. Kojic acid was used as a positive control.

### *Fusarium* strains and culture conditions

Antifungal activities of the defensive secretions emitted by *P*. *hungaricus* and *M*. *unilineatum* were evaluated against seven *Fusarium* species potentially toxigenic and pathogenic for humans and plants: *F*. *avenaceum* (BEOFB810m), *F*. *graminearum* (BEOFB820m), *F*. *incarnatum* (BEOFB830m), *F*. *lateritium* (BEOFB840m), *F*. *proliferatum* (BEOFB850m), *F*. *sporotrichioides* (BEOFB860m), and *F*. *verticillioides* (BEOFB802m). The strains used in the study were determined via ITS and β-tubulin gene sequencing (unpublished results) and maintained in cryovials with 1.5 mL of 30% glycerol stored at -75°C [27] in the culture collection of the Department of Algology, Mycology, and Lichenology, Institute of Botany and Botanical Garden “Jevremovac”, University of Belgrade–Faculty of Biology (BEOFB). Conidial suspensions of selected *Fusarium* species were prepared by washing conidia from the surface of Malt Extract Agar (MEA) slants using sterile saline solution (0.9% NaCl, HemofarmhospitaLogica) with 0.1% Tween 20 (*v/v*). The suspensions were adjusted with sterile saline solution to a final concentration of 1.0 × 10^5^ CFU/mL per the protocol given in Unković et al. [28] and stored at -20°C. Prior to use, 10 μL of conidial suspensions were cultured on solid MEA plates to check validity of the inocula and verify the absence of contamination.

### Anti-*Fusarium* activity assay

To determine the antifungal activity of EtOH, MeOH, Hex, and DCM extracts of P. *hungaricus* and *M*. *unilienatum* defensive secretions against seven *Fusarium* species, the microdilution method described by Hanel and Raether [29] was used in the present study. The test was performed with 96-well microtitre plates (F-bottom, Ratiolab). Different volumes of tested extracts, as well as of antimycotic nystatin (used as a positive control), were dissolved in Malt Extract Broth (MEB) with added 10 μL of inocula per well in order to achieve the required concentrations in the range of from 0.05 to 0.6 mg/mL per well (final volume of 100 μL). The microtitre plates were incubated for 72 h at 25 ± 2°C (UE 500, Memmert), after which the lowest concentrations without apparent growth under a Zeiss Stemi DV4 binocular microscope were designated as minimum inhibitory concentrations (MICs). Minimum fungicidal concentrations (MFCs) were determined by serial subcultivation of 10 μL on microtitre plates with 90 μL of MEB per well and incubation for 72 h at 25 ± 2°C. The lowest concentrations with no visible growth were defined as the MFC values, i.e., the lowest values causing 99.9% inhibition of conidial germination.

### Statistical analysis

Differences in mean values of the antioxidant and antineurodegenerative effects of *P*. *hungaricus* and *M*. *unilineatum* defensive secretion extracts were tested using t-tests, as were differences in mean values of the antioxidant and antineurodegenerative effects of both millipede species and L-ascorbic acid, galantamine, and kojic acid. All statistical procedures were performed in Statistica 7 (StatSoft, Tulsa, OK, USA).

## Results

### Antioxidant activity of tested extracts

Table 1 presents the antioxidant activity of EtOH, MetOH, Hex, and DCM extracts of *P*. *hungaricus* and *M*. *unilineatum* defensive secretions. The tested extracts showed strong antioxidative potentials, which varied depending both on the solvent used for extraction and on the applied assay. The scavenging activities of extracts in the ABTS and DPPH tests in terms of EC_50_ values ranged from 0.114 ± 0.002 to 5.439 ± 0.441 mg/mL. The highest antioxidative capacities were noticed for the MetOH extract of *P*. *hungaricus* defensive secretions in both the ABTS assay (0.972 ± 0.036 mg/mL) and the DPPH assay (0.114 ± 0.002 mg/mL), values which were only 3.7-fold and 2.8-fold lower, respectively, than that of the commercial antioxidant L-ascorbic acid. At the same time, the lowest antioxidative activity was observed for the MetOH extract of *M*. *unilineatum* secretions in both assays (5.439 ± 0.441 mg/mL and 3.451 ± 0.184 mg/mL, respectively). The same trend characterized the observed reducing power capacity, the maximum reducing power being recorded for the MetOH extract of *P*. *hungaricus* secretions (1.003 ± 0.036 mg AAE/mg of dried extract), which was comparable with the reducing power of L-ascorbic acid, while the minimum reducing power was registered for the MetOH extract of *M*. *unilineatum* secretions (0.098 ± 0.012 mg AAE/mg of dried extract) (Table 1).

**Table 1:** Antioxidative activities of ethanol (EtOH), methanol (MetOH), hexane (Hex), and dichloromethane (DCM) extracts of *Megaphyllum unilineatum* (MUN) and *Pachyiulus hungaricus* (PHU) defensive secretions and L-ascorbic acid. Values in bold indicate extracts with a significantly stronger antioxidant effect. Data are expressed as means ± SE.

	DPPH (EC_50_ (mg mL^-1^))			ABTS (EC_50_ (mg mL^-1^))			Reducing power (mg AAE/mg of dried extract)		
Extract	Species		t	Species		t	Species		t
	MUN	PHU		MUN	PHU		MUN	PHU	
EtOH	1.118 ± 0.024	**0.155 ± 0.014**	34.736***	1.502 ± 0.036	**1.122 ± 0.028**	8.291**	0.765 ± 0.033	**0.918 ± 0.022**	3.892*
MetOH	3.451 ± 0.184	**0.114 ± 0.002**	18.121***	5.439 ± 0.441	**0.972 ± 0.036**	10.092***	0.098 ± 0.012	**1.003 ± 0.036**	23.718***
Hex	0.632 ± 0.001	**0.498 ± 0.001**	107.974***	4.117 ± 0.368	**2.422 ± 0.049**	4.564*	0.120 ± 0.007	**0.449 ± 0.014**	24.158***
DCM	0.429 ± 0.002	**0.174 ± 0.002**	87.866***	2.543 ± 0.119	**1.905 ± 0.053**	4.917**	0.454 ± 0.009	**0.591 ± 0.013**	8.619**
L-ascorbic acid	0.041 ± 0.002		EtOH***	0.263 ± 0.011		EtOH***			
			MetOH***			MetOH***			
			Hex***			Hex***			
			DCM***			DCM***			

* P<0.05

** P<0.01

*** P<0.001

### Anticholinesterase and antityrosinase activity of tested extracts

All extracts of the studied species exhibited inhibition of AChE and tyrosinase at a concentration of 100 μg/mL (Table 2). Inhibition of AChE ranged between 67.5 ± 7.9 and 83.0 ± 6.2%, and no statistically significant differences were noted between extracts. The EtOH and Hex extracts of *M*. *unilineatum* secretions showed the strongest inhibition of AChE, approximately 82%, a value which was somewhat lower than that of the commercial inhibitor galantamine (96.1 ± 0.2%). The lowest inhibition rate was observed for the Hex extract of *P*. *hungaricus* secretions (67.5 ± 7.9%) (Table 2). In the case of tyrosinase, strong inhibition (ranging from 43.6 ± 2.7 to 58.5 ± 2.0%) was noted for all of the studied extracts, the maximum level of inhibition being recorded for the Hex extract of *M*. *unilineatum* secretions (Table 2).

**Table 2:** Antineurodegenerative activities of ethanol (EtOH), methanol (MetOH), hexane (Hex), and dichloromethane (DCM) extracts of *Megaphyllum unilineatum* (MUN) and *Pachyiulus hungaricus* (PHU) defensive secretions, galantamine, and kojic acid. Values in bold indicate extracts with a significantly stronger antineurodegenerative effect. Data are expressed as means ± SE.

AChE (Acetylcholinesterase inhibition (%))				Tyr (Tyrosinase inhibition (%))			
Extract	Species		t	Extract	Species		t
	MUN	PHU			MUN	PHU	
EtOH	82.972 ± 6.169	70.075 ± 6.793	1.406^ns^	EtOH	54.848 ± 2.121	53.333 ± 5.043	0.277^ns^
MetOH	72.822 ± 3.521	77.781 ± 4.852	0.827^ns^	MetOH	47.273 ± 1.389	43.636 ± 2.727	1.188^ns^
Hex	81.074 ± 8.972	69.419 ± 11.217	0.811^ns^	Hex	**58.485 ± 1.987**	43.939 ± 1.093	6.414*
DCM	74.878 ± 10.451	67.554 ± 7.951	0.558^ns^	DCM	50.015 ± 1.618	52.630 ± 1.048	1.357^ns^
Galantamine	96.122 ± 0.249		EtOH***	Kojic acid	75.883 ± 0.431		EtOH***
			MetOH***				MetOH***
			Hex***				Hex***
			DCM***				DCM***

^ns^ not significant

* P<0.05

*** P<0.001

### Anti-*Fusarium* activity of tested extracts

The MIC and MFC values of EtOH, MeOH, Hex, and DCM extracts of *P*. *hungaricus* and *M*. *unilineatum* defensive secretions are summarized in Table 3. In both cases, the strongest antifungal activity was observed for EtOH extracts, with MIC and MFC values in the range of from 0.13 to 0.32 mg/mL. For the defensive secretions of *P*. *hungaricus*, the Hex and DCM extracts were shown to possess the weakest antifungal potential, with MIC values in the range of from 0.28 to 0.52 mg/mL and MFCs in the range of from 0.30 to 0.60 mg/mL. The MetOH, Hex, and DCM extracts of *M*. *unilineatum* defensive secretions had roughly the same activity. All of the tested *P*. *hungaricus* and *M*. *unilineatum* extracts showed significantly stronger antifungal activity (>0.60 mg/mL) than that of the commercial antimycotic nystatin, for which MIC and MFC values were not determined.

**Table 3:** Minimum inhibitory concentrations (MICs) and minimum fungicidal concentrations (MFCs) of ethanol (EtOH), methanol (MetOH), hexane (Hex), and dichloromethane (DCM) extracts of *Pachyiulus hungaricus* and *Megaphyllum unilineatum* defensive secretions against seven *Fusarium* species (mg/mL).

Tested microfungi	*Pachyiulus hungaricus* (mg/mL)								*Megaphyllum unilineatum* (mg/mL)								Nystatin (mg/mL)	
	EtOH		MetOH		Hex		DCM		EtOH		MetOH		Hex		DCM			
	MIC	MFC	MIC	MFC	MIC	MFC	MIC	MFC	MIC	MFC	MIC	MFC	MIC	MFC	MIC	MFC	MIC	MFC
*Fusarium avenaceum*	0.15	0.15	0.20	0.20	0.32	0.35	0.30	0.35	0.20	0.27	0.32	0.38	0.47	0.49	0.50	0.58	>0.60	>0.60
*Fusarium graminearum*	0.30	0.44	0.45	0.55	0.48	0.55	0.47	0.58	0.30	0.30	0.50	0.60	0.45	0.55	0.55	0.60	>0.60	>0.60
*Fusarium incarnatum*	0.23	0.37	0.30	0.44	0.52	0.62	0.42	0.55	0.15	0.22	0.36	0.45	0.50	0.60	0.45	0.55	>0.60	>0.60
*Fusarium lateritium*	0.20	0.25	0.27	0.35	0.28	0.38	0.35	0.45	0.13	0.20	0.42	0.54	0.30	0.40	0.30	0.40	>0.60	>0.60
*Fusarium proliferatum*	0.20	0.30	0.22	0.30	0.35	0.45	0.42	0.57	0.15	0.22	0.30	0.36	0.45	0.45	0.45	0.55	>0.60	>0.60
*Fusarium sporotrichioides*	0.26	0.30	0.30	0.30	0.50	0.60	0.50	0.60	0.30	0.30	0.45	0.55	0.55	>0.60	0.50	0.60	>0.60	>0.60
*Fusarium verticillioides*	0.30	0.30	0.35	0.40	0.47	0.55	0.43	0.55	0.30	0.30	0.48	0.58	0.50	0.60	0.50	0.50	>0.60	>0.60

With MIC and MFC values of 0.15 mg/mL, *F*. *avenaceum* was the most susceptible isolate to treatment with the defensive secretion of *P*. *hungaricus*, while *F*. *sporotrichioides* and *F*. *graminearum* were the most resistant. On the other hand, the lowest MIC and MFC values with the defensive secretion of *M*. *uilineatum* were documented for *F*. *lateritium*, while the highest concentrations needed to inhibit and completely suppress growth were recorded for *F*. *sporotrichioides* and *F*. *verticillioides*.

## Discussion

Even though it is widely accepted that plant and fungal extracts are the main natural sources of different bioactive compounds, today many animal species have become a focal point in the search for new biomolecules with promising properties [30,31]. In that regard, many invertebrates, especially arthropods, have been considered a promising source of various compounds capable of reducing the harmful effect of reactive oxygen species (ROS), either as food or as therapeutic products [32,33]. According to available literature, the present paper is the first to treat the antioxidative activity of defensive secretions obtained from juliformian millipedes. In a previous study, evaluation of antimicrobial activity and chemical analysis of the defensive secretion obtained from *P*. *hungaricus* identified the presence of 44 compounds [19]. The majority of these compounds were quinones and pentyl and hexyl esters of saturated and unsaturated fatty acids with chain lengths ranging from C14 to C18. Among them, two quinones were dominant: 2-methyl-1,4-benzoquinone and 2-methoxy-3-methyl-1,4-benzoquinone [19]. Defensive secretions of *M*. *unilineatum*, which show moderate antibacterial activity but have a strong antifungal potential [18], are composed of 38 components. The most dominant of quinones (and of all components) is 2-methoxy-3-methyl-1,4-benzoquinone. The ester fraction is represented with pentyl, hexyl, heptyl, octyl, nonyl, and decyl esters of saturated and unsaturated long-chain fatty acids (C16 and C18-C20). Additionally, two ketones are also present in the defensive secretions of *M*. *unilineatum*: 1-octen-3-one and 3-octanone [18]. This chemical composition is of a great importance in attempting to interpret the antioxidative activity of extracts.

Generally, quinones are an important class of compounds known for their chemical reactivity and important biological functions [34]. The basis for their reactivity lies in the ability to undergo one or two electron reductions (resulting in emergence of the semiquinone or hydroquinone form, respectively). Also, due to their electrophilic character, quinones are subject to nucleophilic attack [34,35]. As already mentioned, julid defensive secretions contain this class of chemical compounds in the form of *p*-benzoquinones. It has been shown that benzoquinones and particularly their hydroxylated derivatives can act as radical scavengers [36]. It has also been demonstrated that a hydroxyl group can be easily attached to the free position of the quinone ring and that at least one methoxy group can be substituted with a hydroxyl group. The obtained hydroxylated species are rather stable under physiological conditions and possess a notable radical-scavenging potential [36].

Hydroquinone and its substituted derivatives also exhibit an antioxidative potential, whether as chain-breaking antioxidants [37,38] or as free radical scavengers [39]. Their high antioxidant activity is determined by their ability to terminate kinetic chains due to reaction with different radicals [38]. Burton et al. [40] showed that the reactivity of substituted phenols increases with augmentation of the electron donating-ability of substituents located in the *p*-position. Thus, hydroquinones in the tested defensive secretions can be seen as another cause of the observed antioxidative effect.

The presence of benzoquinones in large amounts in extracts of defensive secretions might be the crucial reason for the high rate of AChE and tyrosinase inhibition. Several reports demonstrated the potential ability of quinones and their derivatives to inhibit the activity of various enzymes, including AChE [41–43]. It is generally assumed that quinones are highly sulphydryl-reactive compounds and because AChE is an enzyme which contains cysteine residues, it follows that interaction of the inhibitor with sulphhydryl groups of subunits or even those of the enzyme’s active site elucidates the inhibitory influence of benzoquinones [41]. Thus, quinones as highly redox-active molecules can cause loss of protein thiols leading to alteration of protein structure and function [44]. The same principle may be applicable in the case of tyrosinase inhibition.

Apart from quinonic compounds, alkyl esters of various carboxylic acids are present as minor components of the tested extracts. To the best of our knowledge, the detected esters (pentyl, hexyl, heptyl, octyl, nonyl, and decyl esters of unsaturated and saturated long-chain fatty acids [18,19]) have not been previously screened for their antioxidant activity or for their potential ability to inhibit AChE or tyrosinase activity. Although these properties of the detected esters have not yet been confirmed, we think that there is a possibility that these compounds can contribute to the mentioned bioactivities. To be specific, it has been shown that the methyl esters of myristic (C14:0), palmitic (C16:0), stearic (C18:0), oleic (C18:1), linoleic (C18:2), and linolenic (C18:3) acids show antioxidant activities [45,46]. Furthermore, methyl palmitate has been detected as the major component of an extract obtained from the green alga *Scenedesmus intermedius* and accounted for its antioxidant activity [47]. Alkyl esters of long-chain fatty acids can also inhibit AChE and tyrosinase activity. Kissling et al. [48] demonstrated that the ethyl ester of linoleic acid can be a potent inhibitor of AChE activity. The mentioned ester and its methyl congener, as well as methyl and ethyl linolenate, can suppress and inhibit tyrosinase activity [49]. Each registered ester is present in the tested secretions in small percentages, but collectively they constitute 3.4 and 12.7% of the defensive secretions of *M*. *unilineatum* and *P*. *hungaricus*, respectively [18,19]. These data imply that ester components can contribute to the screened bioactivities of the tested extracts. In addition, ketones (1-octen-3-one and 3-octanone) are present in small amounts (0.4 and 0.1%, respectively) in the defensive secretions of *M*. *unilineatum* [18]. Although some ketones can have an antioxidant potential [50], we could not find any data on antioxidant, anti-AChE, or antityrosinase activity of ketones present in the defensive secretions of *M*. *unilineatum*. Based on our results, we think that the mentioned ketones do not contribute (or else their contribution is insignificant) to antioxidant activity (the defensive secretions of *P*. *hungaricus* lack ketones but have stronger antioxidant activity) or to the inhibition of AChE (since there were no statistically significant differences between *M*. *unilineatum* and *P*. *hungaricus* extracts with respect to anti-AChE activity). However, there is a possibility that this class of chemical compounds present in the defensive secretions of *M*. *unilineatum* can enhance antityrosinase activity of the tested secretions, inasmuch as the Hex extract of *M*. *unilineatum* showed a statistically significant difference in the level of inhibition of tyrosinase compared to the extract of *P*. *hungaricus*. This possibility is supported by the fact that some ketones manifest antityrosinase activity [51].

The differences of the antioxidant potential observed between the defensive secretions of *P*. *hungaricus* and *M*. *unilienatum* are correlated with differences in their chemical composition. Although quinones constitute the majority of chemical compounds in both defensive gland exudates, qualitative and quantitative differences are noticeable between them. The defensive secretions of *M*. *unilineatum* do not contain 1,4-benzoquinone, hydroquinone, 2-methoxy-1,4-benzoquinone, and 2-methyl-hydroquinone, while 2-methoxy-5-methyl-hydroquinone, 2,3-dimethoxy-5-methyl-1,4-benzoquinone, and 2,3-dimethoxy-5-methyl-hydroquinone are lacking in the defensive secretions of *P*. *hungaricus*. Of the mentioned differences, the presence of hydroquinone in the defensive secretions of *P*. *hungaricus* can be seen as a cause of their more pronounced antioxidant potential. Hydroquinone is known as a potent antioxidant, and it has also been shown that this chemical compound is a stronger antioxidant than its substituted derivatives [52]. Since esters can have an antioxidant potential [45–47], the higher proportion of them in *P*. *hungaricus* defensive secretions can also contribute to the more pronounced antioxidative activity of their extracts.

The antimicrobial properties of millipede defensive secretions and the possibility of their application as a natural-based replacement for synthetic antimicrobial compounds are still largely unknown, although they are subjects of increasing interest in recent years. However, to the best of our knowledge there have been only three papers published to date on the antifungal and antibacterial potential of defensive secretions obtained from julids and members of the closely related order Spirostreptida. The antifungal potential of the methanol extract of defensive secretions emitted by *P*. *hungaricus* was previously evaluated by Stanković et al. [19]. Growth of eight fungi obtained from medicinal plants was inhibited and completely suppressed by low concentrations of these secretions. The investigated *Fusarium* species (*F*. *subglutinans*, *F*. *semitectum*, and *F*. *equseti*) had MIC and MFC values in the range of from 0.10 to 0.23 mg/mL and 0.13 to 0.25 mg/mL, respectively, which are somewhat lower compared to the methanol extract of *P*. *hungaricus* secretions used in our investigation (MIC 0.20–0.45 mg/mL and MFC 0.20–0.55 mg/mL). In another study, quinone-based defensive secretions of *Orthoporus antillanus* (Diplopoda: Spirostreptidae) were evaluated against several yeasts and filamentous fungi pathogenic for humans and plants. Among the tested microfungi, *F*. *oxysporium*, the causative agent of tomato blight, was particularly susceptible, with growth inhibited at 7.5 μl/mL [53].

The strong antifungal activity of both tested defensive secretions has already been correlated with the presence of quinones in them [18,19]. Although many fungi possess the ability to drive quinone redox cycling and reduce quinones to their less cytotoxic form [54], it is not known how the *p*-benzoquinone-based secretions of millipedes (and other arthropods) achieve an antifungal effect. One possible way may be through the effect of quinones on mitochondrial functions. Haraguchi et al. [55] demonstrated that a substituted *p*-benzoquinone can inhibit the respiratory chain in the yeast *Candida utilis*. Moreover, the same benzoquinone was active against the micromycetes *Mucor mucedo*, *Rhizopus chinensis*, *Aspergillus niger*, and *Penicillium crustosum* [55].

Besides the reactivity of defensive secretion components, the bioactivity of *M*. *unilineatum* and *P*. *hungaricus* extracts can be discussed both from the point of view of the great number of their components and in terms of the presence of different chemical classes in them. The already mentioned study of Haraguchi et al. [55] showed that when one *p*-benzoquinone derivative (2-hydroxy-6-methoxy-3,5-dimethyl-1,4-benzoquinone) alone is tested against different microorganisms, it cannot achieve an antibacterial effect against Gram-positive and Gram-negative bacteria or an antifungal effect against several yeasts. Wahredorf and Wink [56] demonstrated that the combination of different hydroquinones detected in the multi-component defensive secretion of the tenebrioniid *Palembus ocularis* Casey, 1891 was nearly 20 times more pharmacologically active than the individual substances which make up the defensive secretion in question. Also, those authors showed that the addition of a compound from a different chemical class can further increase activity of the tested mixture and that a synergistic effect among different compounds of the same chemical class, as well as a synergistic effect among compounds of different classes, can increase the bioactivity of defensive secretions. This may be the case in the defensive secretions tested by us, since both represent mixtures composed of a great number of compounds (benzoquinones + esters + ketones in *M*. *unilineatum* and benzoquinones + esters in *P*. *hungaricus*).

Exhibiting strong antifungal properties, fluids released from julid ozadenes can be seen as an illustration of semiochemical parsimony (*sensu* Blum [57]). So far, it has been known that millipedes produce and secrete vast assortment of chemical compounds that are utilized as contact toxins, repellents, irritants and/or sedatives [58–61]. These facts give rise to a question–are these primarily defensive secretions to deal with predators which have been co-opted as antifungal agents or vice versa? Recent study on the evolution of complexity of defensive secretions in millipedes [4] can be support for the latter scenario, i.e. millipede defensive secretions are primarily means of antimicrobial protection that can be also used for defense against predators. Additionally, being soil organisms, millipedes are in frequent contact with different microbes that live in the same habitat and that may be or are pathogenic, so there is a possibility that secretions from ozadenes emerged as means of antimicrobial protection. However, an alternative view is that such scenario does not keep up with the diversity of defensive chemicals produced by millipedes (if chemical defense is homologous across Diplopoda) and does not keep up with the mode of releasing of secretions that is triggered by mechanical disturbance and is usually coupled with distinct behavioral attributes such as spiral coiling or contortions of the trunk. Moreover, some millipedes can spray contents of their defensive glands [62]. We are of the opinion that emergence and evolution of millipede defensive secretions can be explained by hypothesis of semiochemical parsimony [57] and that these glandular products possess at least two functions, both antipredator and antifungal protection.

In view of the mentioned roles of secretions in julids, it can be assumed that neutralization of free radicals is not the primary or even a secondary function of these agents. However, the chemical composition of the tested secretions suggests potentially strong antioxidative and antineurodegenerative properties. Additionally, both millipede secretions showed significant antifungal activity against all of the tested *Fusarium* species. We presume that besides the exhibited activities, secretions of the analysed julid species also possess other bioactivities, a possibility which might be the starting point for future studies.
